# Supplementary material for: Green Extraction of Antioxidant Fractions from Humulus lupulus Varieties and Microparticle Production via Spray-Drying
Source: Foods. 2023 Oct 23;12(20):3881. doi: 10.3390/foods12203881 (PMC10667999; doi:10.3390/foods12203881)
Supplement: Supplementary file 1 [file foods-12-03881-s001.zip › foods-2670479-supplementary.pdf]

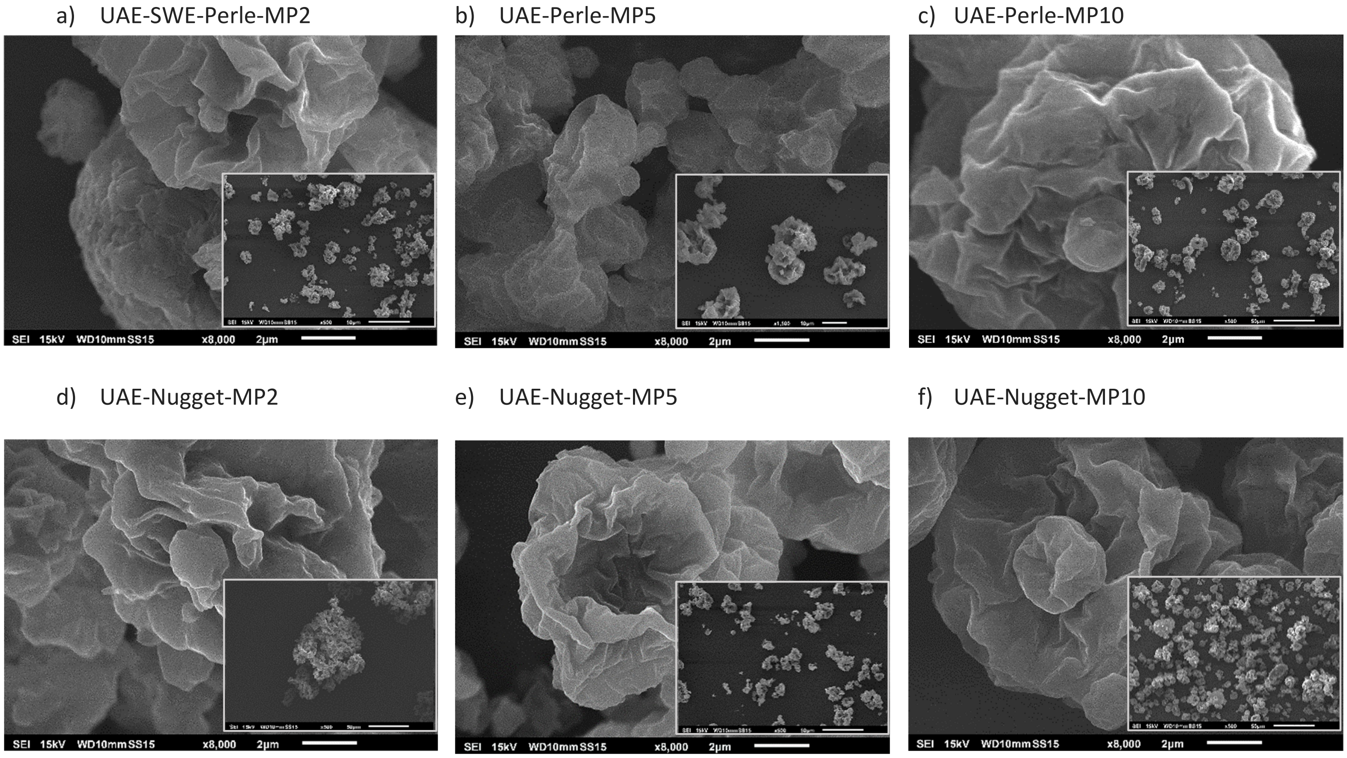

Figure S1. Scanning electron microscopy images of polymeric microparticles formulated with mannitol and the extracts obtained by ultrasound assisted extraction (UAE) for Perle and Nugget varieties from *H. lupulus*. Note: MP2, MP5 and MP10 mean microparticles with mannitol at 2%, 5% and 10% (w/w), respectively. The magnitudes of the images are x8,000 and small image x500.
